# Glutamine regulates ovarian cancer cell migration and invasion through ETS1

**DOI:** 10.1016/j.heliyon.2021.e07064

**Published:** 2021-05-31

**Authors:** Parash Prasad, Sib Sankar Roy

**Affiliations:** a Cell Biology and Physiology Division, CSIR-Indian Institute of Chemical Biology, 4 Raja S. C. Mullick Road, Kolkata 700032, India; b Academy of Scientific and Innovative Research, CSIR- Indian Institute of Chemical Biology Campus, 4 Raja S. C. Mullick Road, Kolkata 700032, India

**Keywords:** Glutamine, MMP, ETS1

## Abstract

Cancer cells are dependent on glutamine for their metabolism and growth. Despite being the most abundant amino acid in the blood, glutamine deprivation occurs in the core of the tumor rendering less access to glutamine to the nearby tumor cells. Tumor cells mostly use the glutamine for mitochondrial oxidative phosphorylation (OXPHOS) to produce energy and the ingredients of the biomass required for the highly proliferating and metastatic ovarian cancer cells. But there is a lack of reports on the regulation of glutamine starvation on metastatic behavior and epithelial to mesenchymal transition (EMT) of ovarian cancer cells. We found that glutamine starvation reduced the migration and invasion properties of the ovarian cancer cells, PA1 and SKOV3. The expression of the invasion-inducing proteins, like matrix metalloproteinases (MMP2 and MMP9), were downregulated upon glutamine starvation. MMP genes are mostly regulated by the ETS1 oncogenic transcription factor in invasive tumor cells. Here we demonstrated the significant involvement of ETS1 on EMT and invasion in glutamine-deprived cells. We have further shown that the regulation of ETS1 expression and nuclear localization upon glutamine starvation is controlled in a cell type-specific manner. In PA1 cells, glutamine-induced ETS1 over-expression is HIF1α-dependent, while in SKOV3, its translocation to the nucleus is regulated through the mTOR pathway. Considering all, our study suggests that glutamine plays a very significant role in migration and invasion in ovarian cancer cells and ETS1 plays a key role in inducing such oncogenic parameters.

## 1 Introduction

Invasion and metastasis in cancer cells are the major steps in oncogenesis. As cancer progression occurs, the tumor cells move from the primary site to a new secondary site. During migration, the cancer cells undergo epithelial to mesenchymal transition (EMT) as the mesenchymal cells are more motile in nature [1]. The actin polymerization and its turnover at the leading edge in such cells are important to facilitate migration. Here the mitochondria provide ATP to fuel the actin rearrangement [2]. The formation of actin stress fibers is most frequently observed in the migrating cells [3]. Apart from ATP-dependant actin-cytoskeleton rearrangement, several other factors are also important for invasion and metastasis. For invasion through the basement membrane in a solid tumor, the cells need to digest the extracellular matrix of the surrounding tissue or the secondary tissue site. This is performed by different matrix metalloproteinases (MMPs) secreted by the tumor cells as their activities are enhanced in solid tumors [4]. These *MMP* (*MMP2 and MMP9*) genes are transcriptionally regulated by different transcription factors, but most importantly ETS1 has been proposed as a promising factor in this regard especially in epithelial ovarian cancer [5]. Upon receiving the microenvironmental stimuli, ETS1 expression increases and it translocate to the nucleus, where it transcriptionally activates different *MMP* genes as well as regulates different genes associated with metabolic reprogramming of tumor cells. Vimentin and VEGF have been reported as Epithelial to Mesenchymal (EMT) markers. Vimentin helps directly in cell motility, whereas VEGF overexpression or treatment found to be elicit EMT in cancer cells [6, 7, 8].

Cancer progression is highly dependent on the metabolic activities of the tumor cells, which are influenced by the availability of two essential nutrients, glucose and glutamine in the cell as well as in the microenvironment. Glutamine is not an essential one for the normal tissues and cells, but the tumor cells consume excess glutamine *in vivo,* as well as *in vitro* for their proliferation. c-Myc and k-Ras mutation found to be involved in the glutamine addiction of the cancer cells [9]. Inhibiting glutamine metabolism or glutamine withdrawal from the media reduces the cell proliferation of the highly metastatic ovarian cancer cells [10]. Although the mechanism behind such proliferation is not well established, glutamine has been shown to activate the STAT3 pathway for the proliferation of these highly metastatic ovarian cancer (OVCA) cells [11]. Further, glutamine has also been reported to activate the mTORC1 pathway to facilitate proliferation [12]. But whether or how glutamine is associated with EMT and invasive behavior following the proliferation of the tumor cells has not been adequately studied and needs investigation.

While attending this significant area of research on ovarian cancer, our findings suggest that the PA1 and SKOV3 cancer cells lose their migratory and invasive potential upon glutamine starvation. In the present report, we have shown the possible mechanism behind these effects.

## 2 Methods

### 2.1 Key resources table

This table contains the list of the materials and resources used to perform the experiments and will be useful to reproduce the same.

**Table:** 

Reagent	Resource	Identifier
**Antibodies**		
Rabbit polyclonal anti-ETS1	Cell Signaling Technology	Cat# 6258BC
Rabbit polyclonal anti-MMP2	Cell Signaling Technology	Cat# 4022, RRID:AB_2266622
Rabbit polyclonal anti-p-AKT	Cell Signaling Technology	Cat# 9271, RRID:AB_329825
Rabbit monoclonal anti-α-TUBULIN	Cell Signaling Technology	Cat# 2125, RRID:AB_2619646
Rabbit polyclonal anti-β-ACTIN	Cell Signaling Technology	Cat# 4967, RRID:AB_330288
Horse anti-rabbit HRP tagged secondary antibody	Cell Signaling Technology	Cat# 7074, RRID: AB_2099233
Goat anti-rabbit IgG Alexa-Fluor 488-labeled secondary antibody	Molecular Probes (Invitrogen)	Cat# A-11008, RRID:AB_143165
Goat anti-rabbit IgG Alexa-Fluor 568-labeled secondary antibody	Thermo Fisher Scientific	Cat# A-21069, RRID:AB_2535730
**Chemicals, Peptides, and Recombinant Proteins**		
Rapamycin	Calbiochem	Cat# 553210; CAS: 67-68-5, 53123-88-9
HIF1α inhibitor	Calbiochem	Cat# 400089; CAS: 329932-55-0
Gelatin	Sigma-Aldrich	Cat# G2500; CAS: 9000-70-8
DAPI	SRL	Cat# 18668; CAS: 28718-90-3
Rhodamine tagged phalloidin	Invitrogen	Cat# R415
SYBR green	Bio-Rad	Cat# 1725120
RNAiso Plus	TAKARA Bio	Cat# 9109
**Experimental models: cell lines**		
Human: PA1	ATCC	Cat# CRL-1572, RRID:CVCL_0479
Human: SKOV3	ATCC	Cat# HTB-77, RRID:CVCL_0532
**Critical commercial assays**		
iSCRIPT cDNA synthesis kit	Bio-Rad	1708891
Nuclear-cytoplasmic isolation kit	Thermo Scientific	Cat# 78833
**Cell culture reagents**		
MEM-α	HIMEDIA	Cat# AL080A
MEM- α (W/O GLN)	HIMEDIA	Cat# AL080
RPMI-1640	HIMEDIA	Cat# AL199A
RPMI-1640 (W/O GLN)	HIMEDIA	Cat# AL060
**Oligonucleotides**		
Primer for *ETS1* (Forward: GGAGGACCAGTCGTGGTAAA; Reverse: AACTGCCATAGCTGGATTGG)	[13]	NA
Primer for *MMP2* (Forward: TGATCTTGACCAGAATACCATCGA; Reverse: GGCTTGCGAGGGAAGAAGTT)	[13]	NA
Primer for *MMP9* (Forward: ACCTCGAACTTTGACAGCGAC; Reverse: GAGGAATGATCTAAGCCCAGC)	[13]	NA
Primer for *VIM* (Forward: ACACCCTGCAATCTTTCAGACA; Reverse: GATTCCACTTTGCGTTCAAGGT)	[14]	NA
Primer for *VEGF* (Forward: CTGGAGCGTTCCCTGTGGGC; Reverse: CCTTCCTCCTGCCCGGCTCA)	This paper	NA
Primer for *L19* (Forward: GCGGATTCTCATGGAACACA; Reverse: GGTCAGCCAGGAGCTTCTTG)	This paper	NA

### 2.2 Cell culture and treatments

PA1 (ATCC Cat# CRL-1572, RRID:CVCL_0479) cells were cultured in MEM (α modification, GIBCO) media and SKOV3 (ATCC Cat# HTB-77, RRID:CVCL_0532) cells were cultured in RPMI-1640 (GIBCO) media supplemented with 10% FBS (GIBCO). PA1 cell line was authenticated by STR profile analysis and the SKOV3 cells were cultured for less than 15 passages, therefore STR profiling was not performed for this cell line. The cells were maintained in a 5% CO_2_ and 37 °C humidified incubator. Passaging of the cells was done by decanting the medium followed by trypsinization (0.25%, GIBCO) of the cells and then centrifuged in 1000 g for 5 min. After decanting the supernatant, the cells were resuspended in a complete medium and seeded in a cell plate for treatment. Splitting was done when cells attain about 80% of confluency in the T25 flask and seeded at 0.5 × 10^6^ cell/35 mm dish for PA1 (0.3 × 10^6^ cell/35 mm dish for SKOV3). For treatment after reaching 60% confluency, the cells were first starved with an incomplete medium (1% FBS) for 4–6 h and then treated with either 2 mM glutamine supplemented or glutamine-free complete medium (10% FBS). HIF1α inhibitor (Calbiochem, Cat # 400089) and Rapamycin (Calbiochem, Cat # 553210) was given at the onset of glutamine re-supplementation. After the completion of treatment, the pH of the conditioned medium was directly measured with a pH meter (Sartorius).

### 2.3 Quantitative real-time PCR

Total RNA was isolated from the cultured cells (about 1 × 10^6^ cells for PA1 and 0.8 × 10^6^ cells for SKOV3) grown in 35 mm cell culture dish, using RNAiso Plus (TAKARA Bio, Cat #9109) following a standard protocol [13]. The RNA concentration was obtained by directly measuring the absorbance of the resuspended RNA pellet (1:300 in DEPC treated water) at 260 nm wavelength. RNA integrity and purity were confirmed by direct observation of 1% agarose gel loaded with 1 μl RNA and 260 nm/280 nm absorbance ratio. cDNA synthesis was done with 500 ng of isolated RNA using iScript cDNA synthesis kit (Bio-Rad, Cat #1708891). Relative expression of the genes was measured by real-time qPCR (Applied Biosystems, 7500 Real-Time PCR Software, RRID: SCR_014596) using iTaq universal SYBR green supermix (Bio-Rad, Cat #1725120), and normalized to L19 mRNA levels [15]. The real-time PCR thermal profiles are: initial denaturation (95 °C for 20s) then the 40 cycling steps (95 °C for 15s, annealing for 30 s at 60 °C, extension at 72 °C for 30s) followed by melting curve analysis stage (95 °C for 15s, 60 °C for 60s, 95 °C for 15s and 60 °C for 15s.). The comparative CT value (^ΔΔ^CT) was used to measure the relative gene expression. Relative fold changes of genes by qPCR are shown in comparison with control cells with no treatment. The primers were designed using Primer Express software (Primer Express Software, RRID: SCR_017376) and then verified by Primer-Blast (Primer-BLAST, RRID: SCR_003095). Custom oligos were purchased from IDT (Integrated DNA Technology). The primer sequences are given in Table 1.

**Table 1:** Sequence of primers used for this work.

Gene name	Forward primer (5′–3′)	Reverse primer (5′–3′)	Amplicon size (nt)	Source
*ETS1*	GGAGGACCAGTCGTGGTAAA	AACTGCCATAGCTGGATTGG	304	[13]
*MMP2*	TGATCTTGACCAGAATACCATCGA	GGCTTGCGAGGGAAGAAGTT	90	[13]
*MMP9*	ACCTCGAACTTTGACAGCGAC	GAGGAATGATCTAAGCCCAGC	133	[13]
*VIM*	ACACCCTGCAATCTTTCAGACA	GATTCCACTTTGCGTTCAAGGT	80	[14]
*VEGF*	CTGGAGCGTTCCCTGTGGGC	CCTTCCTCCTGCCCGGCTCA	180	This paper
*L19*	GCGGATTCTCATGGAACACA	GGTCAGCCAGGAGCTTCTTG	68	This paper

### 2.4 Fluorescence microscopy

The cells were cultured on glass coverslips (Blue Star 22 × 22 mm square No. 1 cover glass sterilized with 70% methanol) and were given treatment after serum starvation. After discarding the medium, the cells were fixed with 4% paraformaldehyde (pH 7.4) for 20 min followed by permeabilization with 0.1% Triton X-100 for 15 min at room temperature. Then the fixed cells were incubated overnight with anti-ETS1 antibody (1:200; Cell Signaling Technology Cat# 6258BC, Lot # 1) in 4 °C after 1 h of blocking in 3% BSA in PBS (HIMEDIA, India, Cat# TL1101) solution. Then the cells were stained with either Alexa-Fluor 488-labeled secondary antibody (1:400; Invitrogen, Cat# A11008, Lot# 2051237) or Alexa-Fluor 568-labeled secondary antibody (1:400; Invitrogen, Cat# A-21069, Lot# 108193) for 2 h in room temperature and the nucleus was stained with DAPI (0.25 μg/ml) for 5 min. To stain the filamentous actin, after permeabilization cells were incubated with Rhodamine-phalloidin (Cat# R415, Invitrogen) for 20 min in room temperature. Images were taken using TCS SP8 confocal microscope (Leica Microsystems, RRID: SCR_008960) with 63X oil immersion objective lens and Olympus BX51 microscope (Olympus, RRID: SCR_017564) with 40X objective lens.

### 2.5 *In vitro* Matrigel invasion assay

The Matrigel invasion chamber was purchased from BD Biosciences (Cat # 354480, 354481) and the manufacturer's protocol was followed. Briefly, the untreated cells and the glutamine starved cells (SKOV3 cell passage no. 11) were trypsinized separately, their number was counted and plated on the upper chamber. Then the cells were allowed to invade for 22 h. The seeding cell number was kept according to the manufacturer's protocol. After crystal violet staining and followed by methanol fixation, the invaded cells were observed and counted under an inverted microscope (EVOS, Thermo Fischer Scientific).

### 2.6 *In vitro* wound healing assay

The cells were seeded in 35 mm cell culture dishes and treated with complete medium with or without glutamine for 24 h at approximately 70% confluency. Then the cells were scratched with a 200 μl microtip through the diameter of the culture dishes and the migration of the cells towards the wound was detected at 24 h intervals under an inverted microscope (EVOS, Thermo Fischer Scientific). The wound width was measured with the help of ImageJ software (ImageJ, RRID: SCR_003070, version # 1.50i). After opening the image in ImageJ, the scale bar pixel: size ratio of the image was fed to the software. Then a horizontal line was drawn through the wound width and the length of the line was acquired with the 'measure' option under the 'analyze' tab of ImageJ toolbar. 3–4 horizontal lines were drawn through the wound and an average was taken for each Image.

### 2.7 Western blot analysis

The cultured cells were lysed and the protein extraction was performed as described previously [13]. Lysates were immunoblotted with antibodies specific for ETS1 (Cell Signaling Technology, Cat# 6258BC, Lot# 1), α-tubulin (Cell Signaling Technology Cat# 2125, RRID:AB_2619646, Lot# 11), β-actin (Cell Signaling Technology Cat# 4967, RRID:AB_330288, Lot# 6), MMP2 (Cell Signaling Technology Cat# 4022, RRID:AB_2266622, Lot# 2) and p-AKT (Cell Signaling Technology Cat# 9271, RRID:AB_329825, Lot# 12). The chemiluminescent bands were detected using horseradish peroxidase-labelled secondary antibodies (1:5000) (Cell Signaling Technology Cat# 7074, RRID: AB_2099233, Lot# 28) and ECL luminescence substrate (Bio-Rad, Cat# 170-5061). The expression of proteins was normalized with α-tubulin or β-actin.

### 2.8 TCGA data acquirement

The TCGA (The Cancer Genome Atlas) processed data were acquired from www.oncolnc.org and the Kaplan plot for *ETS1* expression in ovarian cancer patients was generated with the lower and upper percentile of 50:50 (total number of patients is 294).

### 2.9 Gelatine zymography

After glutamine starvation, 2 ml of the conditioned medium was collected, frozen, and lyophilized. Then the lyophilized sample was dissolved in 200 μl of distilled water. Then protein concentration was measured and about 100 μg of protein was loaded per well in 0.1% gelatine-10% polyacrylamide gel. No SDS was used in the preparation of protein loading buffer and gel. After running, the gel was washed with 2.5% Triton X-100 and incubated in digestion buffer (NaCl 0.2 M, CaCl_2_ 4.5 mM, Tris 50 mM) for overnight in 37 °C. Then the gel was stained with 0.1% Coomassie brilliant blue for 2 h and the image was captured after washing.

### 2.10 Statistical analysis

All the statistical significance was calculated with values acquired from at least three biological replications of an experiment. The acquired data were expressed as the mean ± SEM. The two-way ANOVA (followed by Bonferroni post-test analysis) was performed, where the number of variables was 2. The statistical significance was estimated by two-tailed Student's t-test. p-value<0.05 was considered as statistically significant (∗) and p-value<0.001 were considered to be highly statistically significant (∗∗). All statistical analyses were performed using GraphPad Prism-5 (GraphPad Prism, RRID: SCR_002^7^98) software.

## 3 Results

### 3.1 Glutamine starvation reduces migratory and invasive potential of ovarian cancer cells

**Figure 1:**
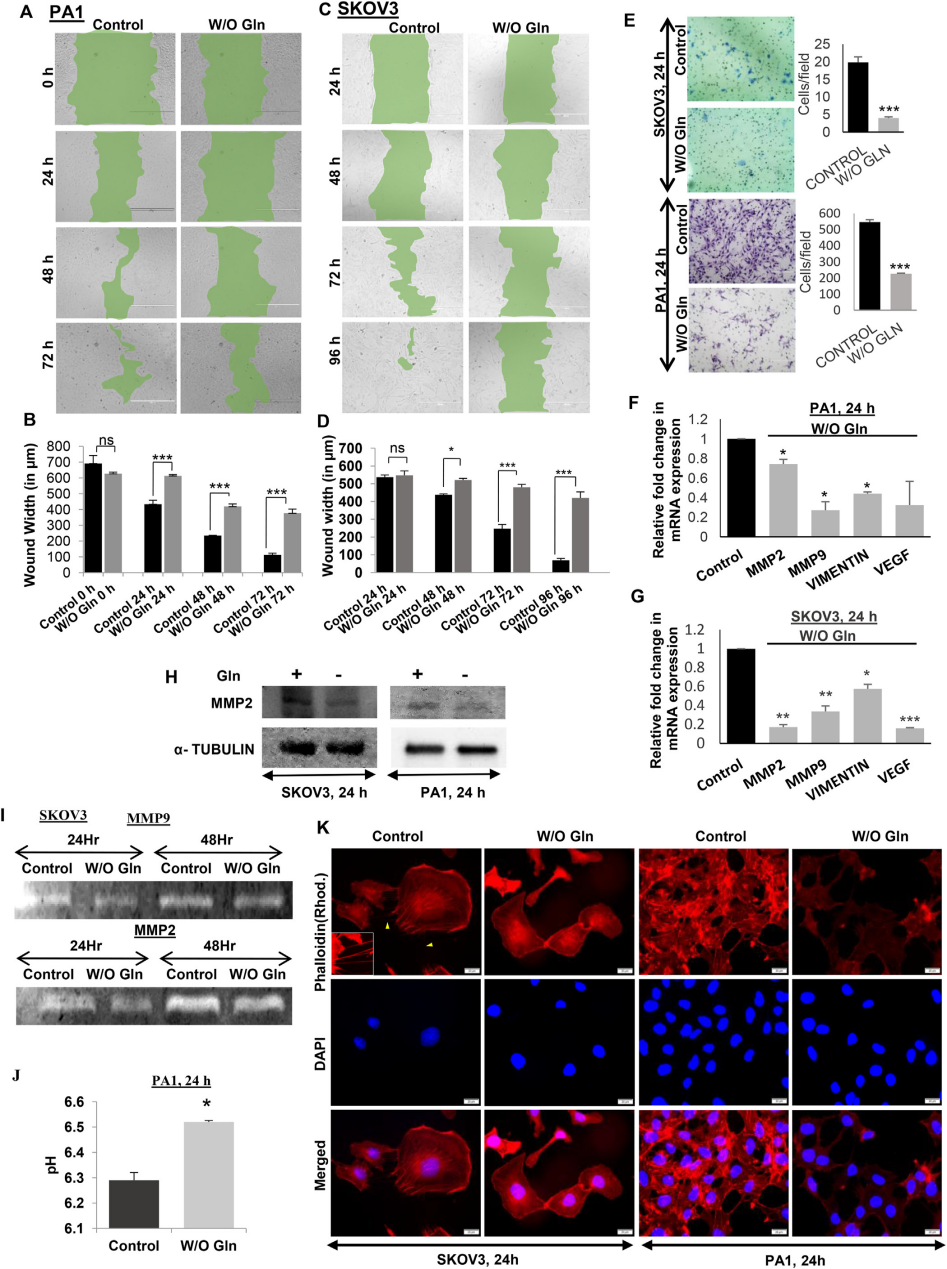
Glutamine starvation reduces migration and invasion capacity of cancer cells. Wound healing assay of PA1 (A, B) and SKOV3 (C, D) cells reveals reduced healing in absence of glutamine (n = 3). SKOV3 and PA1 cells showed reduced invasion capacity in absence of glutamine (E) (n = 3). mRNA expression of MMP2, MMP9, Vimentin, and VEGF reduced in PA1 cells in absence of glutamine (F) (n = 3). Reduced mRNA expression of MMP9 and Vimentin was observed in SKOV3 cells in absence of glutamine (G) (n = 3). MMP2 protein expression was reduced after glutamine starvation (H). Gelatine zymography shows that MMP2 and MMP9 activity was reduced after glutamine starvation (I). High extracellular pH in absence of glutamine in PA1 cells (J) (n = 3). Phalloidin staining revealed the reduced actin stress fiber formation in SKOV3 and PA1 cells in absence of glutamine (K). Data are expressed in ±SEM, and statistical significance was calculated using two-tailed Student's t-test and the two-way ANOVA was performed followed by Bonferroni post-test analysis (A–D). (∗p < 0.05, ∗∗p < 0.01). ns is statistically non-significant. Scale bars 400 μm (A, C, E) and 20 μm (K).

Migration and invasion are the two basic criteria for increased metastasis of cancer cells. To check the changes in the migration and invasion properties of the cells, we performed wound healing assay and found that glutamine starved cells failed to heal the wound in PA1 (Figure 1A, B) and SKOV3 (Figure 1C, D) cells. The Matrigel invasion assay showed that a smaller number of cells could migrate upon glutamine starvation (Figure 1E). Consequently, the mRNA expression of *MMP2* and *MMP9* genes and EMT markers like *VIMENTIN* and *VEGF* were also reduced significantly (Figure 1F–G). Similarly, the MMP2 protein expression as well as MMP2 and MMP9 in-gel activity was reduced after glutamine starvation (Figure 1H–I, Supplementary Figure 1–3). The activity of MMPs is dependent upon the extracellular pH [16] and the pH was found to be significantly high in absence of glutamine (Figure 1J) in the culture medium of PA1 cells. This indicates that glutamine withdrawal significantly reduced the migration and invasion of the ovarian cancer cells by reducing the activities of MMPs. During cell migration, the actin cytoskeleton helps in the formation of new lamellipodium. The actin cytoskeleton interacts with myosin and forms the actin stress fibers, which helps in pushing the cell body mass to the forward direction.

In accordance with our previous data, we have found that there was a reduction in the actin fiber formation in absence of glutamine (Figure 1K). Actin polymerization and stabilization supports the formation of inter-cellular tunneling nanotubules, which are prominent in untreated SKOV3 cells (arrowheads in Figure 1K) but are undetectable in glutamine starved cells.

### 3.2 ETS1 regulates the expression of MMPs and is associated with a poor prognosis of ovarian cancer

**Figure 2:**
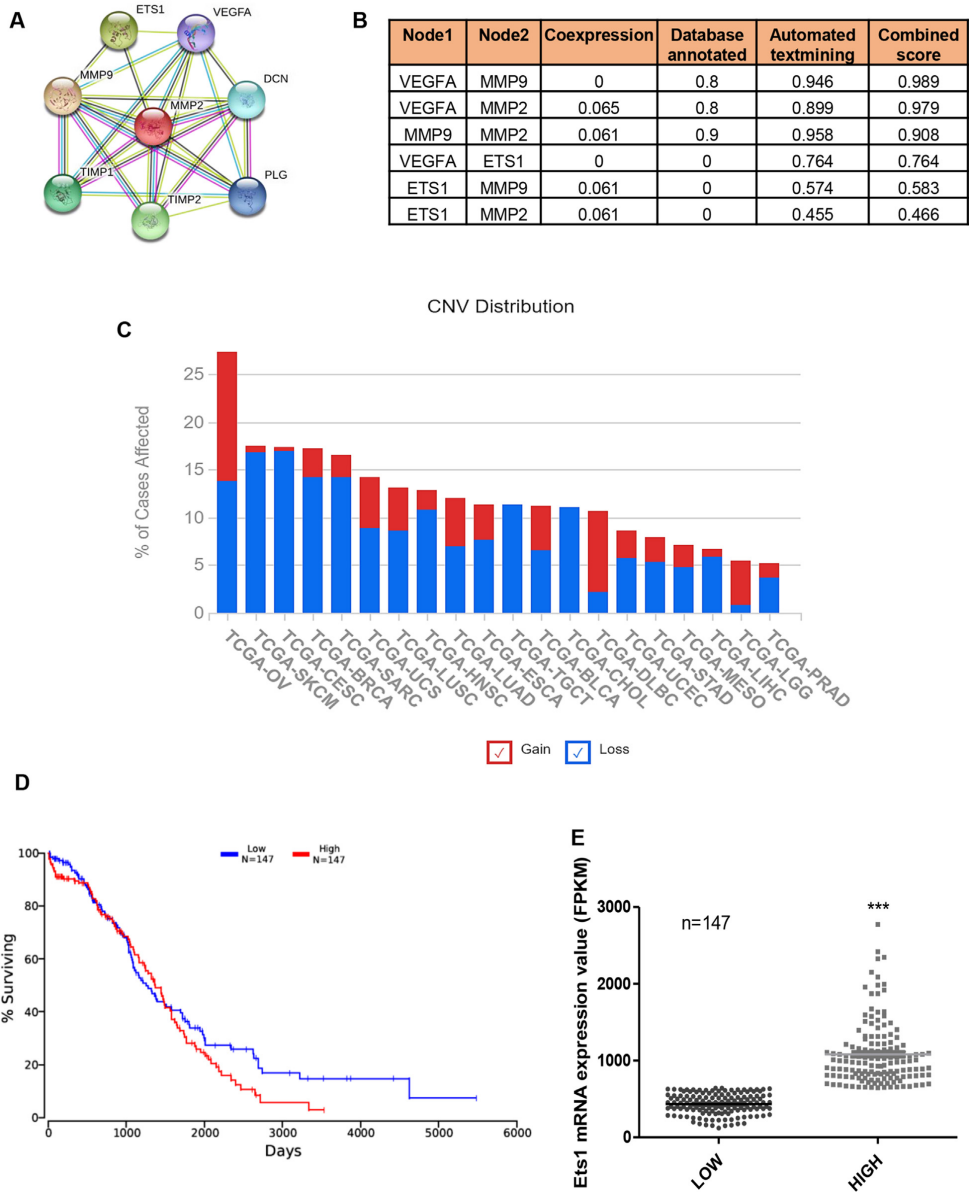
ETS1, the regulator of MMPs, is strongly associated with a poor prognosis of ovarian cancer. STRING interactome analysis showed ETS1 as a common interactor with MMP2, MMP9 and VEGFA (A, B). TCGA data showing different cancers affected with ETS1 gain and loss of function (C). Kaplan plot showing survival percentage of patients expressing high and low levels of ETS1 (D). Dot plot of mRNA expression of ETS1 of different patients (E) (n = 147). Data are expressed in ±SEM, and statistical significance was calculated using two-tailed Student's t-test (∗p < 0.05, ∗∗p < 0.01, ∗∗∗p < .001).

To find the possible regulating factor for the expression of MMP2, MMP9, vimentin, and VEGF, the STRING [17] database was used. We found the oncogenic transcription factor ETS1 as a potent regulator of MMP2, MMP9, and VEGF, which were altered in the absence of glutamine, with a significant score (Figure 2A–B). To find the importance of ETS1 in ovarian cancer, we performed TCGA data mining and found that relative to other cancers, ovarian cancer is most affected by ETS1 gain of function (Figure 2C). Patients with high ETS1 also show low survival probability (Figure 2D). The mRNA expression of ETS1 between the compared low and high group set was significantly different (Figure 2E) with a positive cox regression coefficient value of 0.039 as acquired from OncoLnc [18].

### 3.3 ETS1 expression and translocation are regulated through glutamine

**Figure 3:**
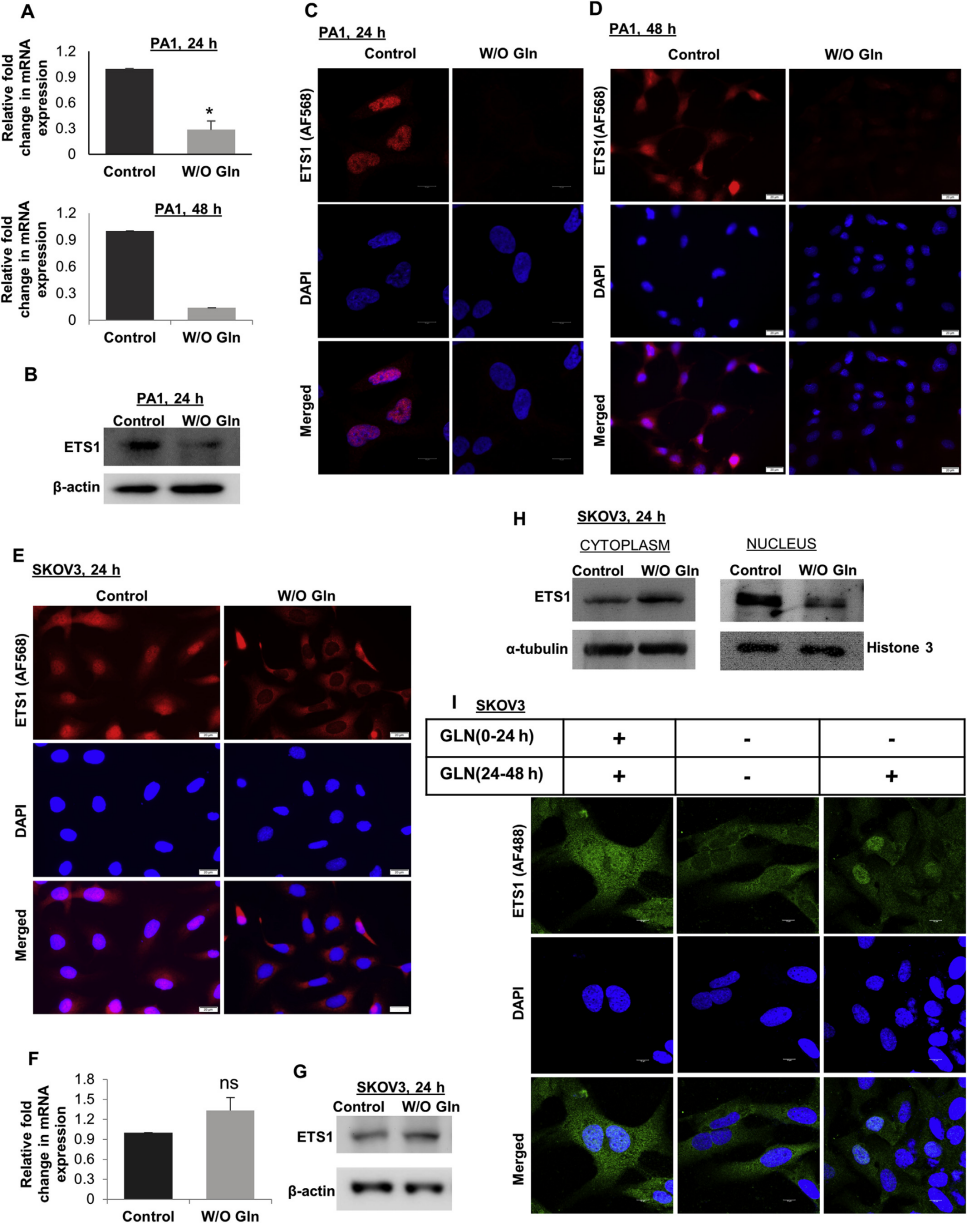
Glutamine regulates ETS1 expression. ETS1 expression was reduced after 24 h (n = 3) and 48 h (n = 2) of glutamine starvation in PA1 cells in mRNA (A) and protein level (B). Microscopy data also reveals a reduction in ETS1 expression in PA1 cells after glutamine starvation (C, D). In SKOV3 cells glutamine starvation cause translocation of the ETS1 from the nucleus to the cytoplasm (E). There is no change in expression of ETS1 in mRNA (F) (n = 3) and protein level (G) in SKOV3 cells in absence of glutamine. Western blot analysis of cytoplasm and nuclear protein fraction revealed translocation of ETS1 from the nucleus to the cytoplasm in glutamine-starved SKOV3 cells (H). Microscopic data showed reintroduction of glutamine to the glutamine-starved cells causes ETS1 re-translocation to the nucleus (I). Data are expressed in ±SEM, and statistical significance was calculated using a two-tailed Student's t-test (∗p < 0.05, ∗∗p < 0.01). ns is statistically non-significant. Scale bars 10 μm (E, K) and 20 μm (F, G).

ETS1 is also an established regulator of the MMP gene expression [13]. We found that the expression of ETS1 was reduced in mRNA and protein levels after 24 h and 48 h of glutamine starvation in PA1 cells (Figure 3A–B, Supplementary Figure 4). Immunofluorescence microscopy also showed reduced ETS1 protein expression in absence of glutamine in PA1 cells (Figure 3C–D). However, in SKOV3 cells, there was delocalization of ETS1 from the nucleus to the cytoplasm upon glutamine starvation (Figure 3E) without a change in mRNA and protein expression (Figure 3F–G, Supplementary Figure 5). Further, there was a lower amount of ETS1 in the nuclear fraction of glutamine starved cells than the control cells (Figure 3H, Supplementary Figure 6). Glutamine re-supplementation to the 24 h glutamine starved cells were able to re-localize the ETS1 protein to the nucleus. Some cells showed distinct nuclear localization, whereas some had uniformed distribution in the cytoplasm and nucleus, depicting the initiation of nuclear transport (Figure 3I). This indicates that the translocation of ETS1 is glutamine dependent in SKOV3 cells. However, in PA1 cells the expression of ETS1 is reduced upon glutamine starvation.

### 3.4 HIF1α regulates the expression of ETS1, but its localization is regulated through mTOR

**Figure 4:**
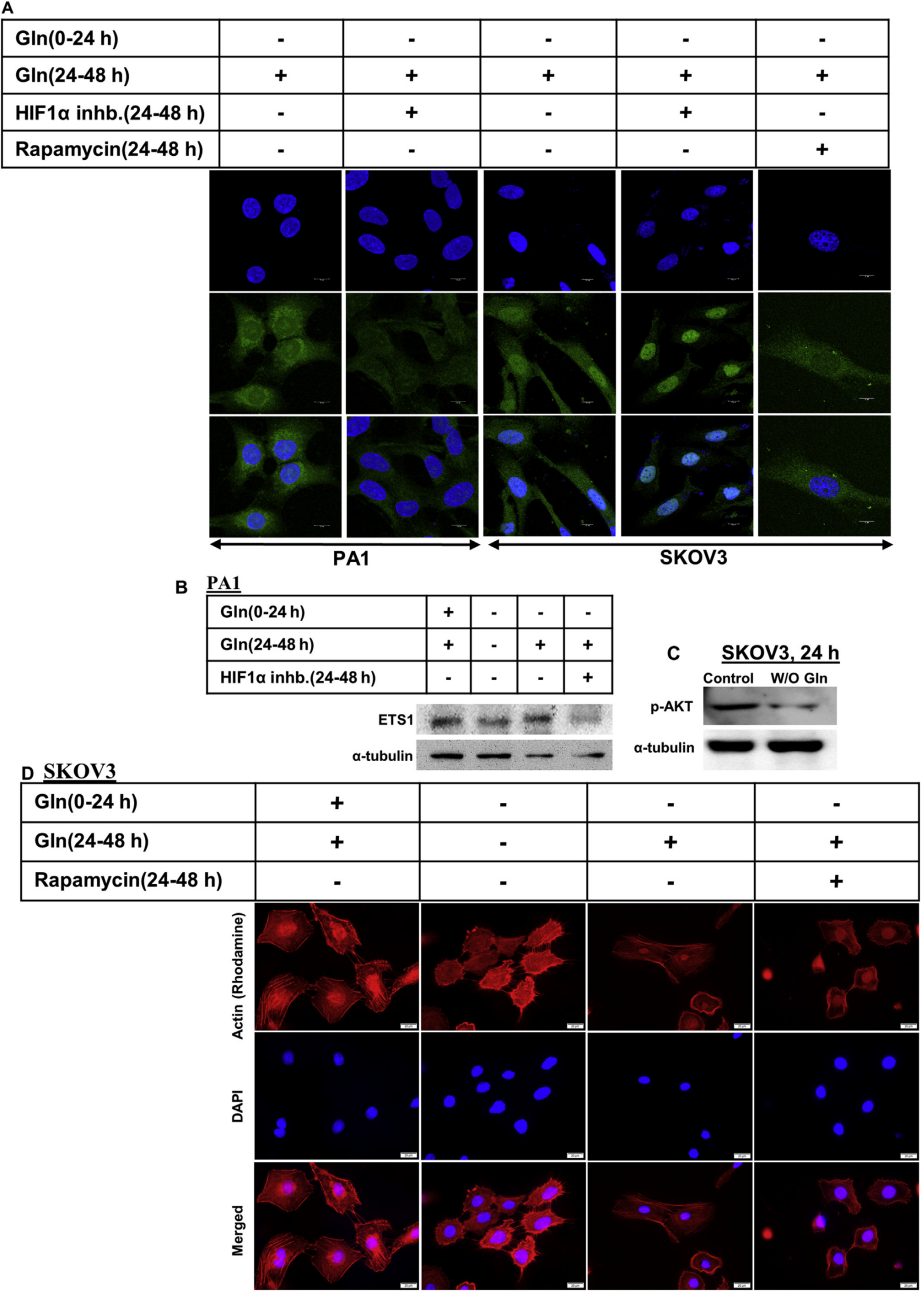
HIF1α and mTOR regulate ETS1 expression and translocation respectively. HIF1α inhibitor successfully inhibited the increase in ETS1 expression upon glutamine reintroduction in glutamine starved PA1 cells (A, B) but failed to inhibit ETS1 translocation in SKOV3 cells. Rapamycin treatment inhibited ETS1 translocation upon glutamine supplementation (A). Glutamine starvation reduces AKT phosphorylation in SKOV3 cells only (C). Rapamycin treatment inhibits actin stress fiber formation in SKVO3 cells after glutamine supplementation (D). Scale bars 10 μm (A) and 20 μm (D).

**Figure 5:**
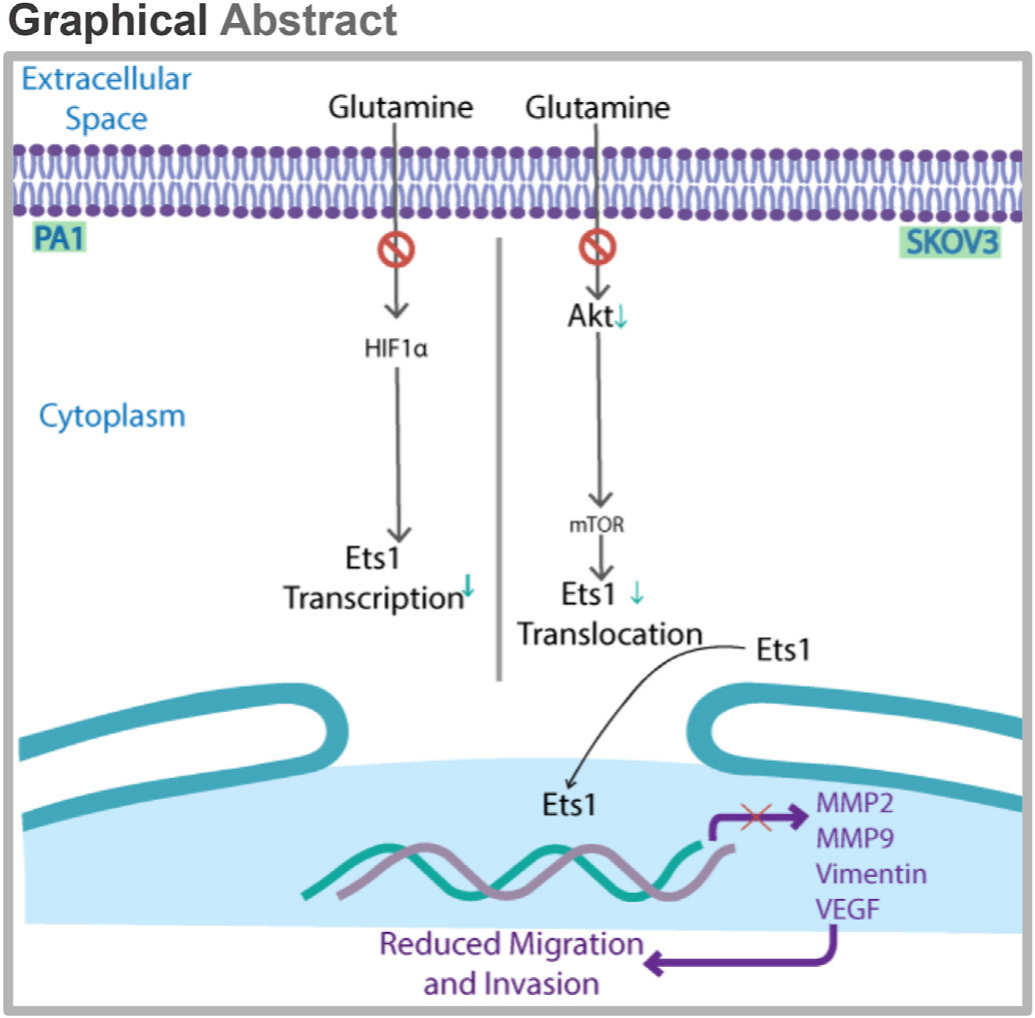
Graphical Abstract. In PA1 cells glutamine starvation reduces ETS1 expression and re-addition of glutamine increases ETS1 expression in HIF1α dependent manner. While in SKOV3 cells, ETS1 translocate to cytoplasm from the nucleus upon glutamine starvation. Glutamine addition increases the nuclear translocation of ETS1 in mTOR dependent manner.

Hypoxia-inducible factor1α (HIF1α) is another important factor, known to regulate EMT and metastasis in tumor cells [19]. According to the earlier data, HIF1α regulates the expression of the ETS1 gene in PA1 ovarian cancer cells [13]. We checked if glutamine-mediated modulation of expression and localization of ETS1 is HIF1α dependent or not. Microscopic studies revealed that when glutamine was supplemented to the glutamine-starved PA1 cells, ETS1 expression was increased, however, the addition of HIF1α inhibitor could successfully inhibit this ETS1 resurrection (Figure 4A–B, Supplementary Figure 7). However, it failed to inhibit the translocation of ETS1 from the cytoplasm to the nucleus in SKOV3 cells (Figure 4A). This signifies that HIF1α is an important factor for the ETS1 expression in PA1 cells, but not in SKOV3, as treating the latter with HIF1α inhibitor did not decrease the ETS1 expression. We have also found that glutamine starvation reduced AKT phosphorylation (Ser 473) in SKOV3 cells (Figure 4C, Supplementary Figure 8). Further, according to previous reports, glutamine induces mTORC1 activity. Interestingly, we found that treating the SKOV3 cells with Rapamycin (inhibitor of mTOR, a direct substrate of Akt) significantly reduced the ETS1 translocation to the nucleus upon glutamine starvation in these cells (Figure 4A). Glutamine starvation reduces actin stress fiber formation in SKOV3 cells and it was regained when glutamine starved cells are supplemented with glutamine. Whereas Rapamycin treatment also reduced the actin stress fiber formation, when glutamine-starved SKOV3 cells were supplemented with glutamine (Figure 4D). Therefore, mTOR seems to be a regulator of cell migration and ETS1 translocation in SKOV3 cells (see Figure 5).

## 4 Discussion

Cancer is regarded as a metabolic disorder considering the involvement with various metabolic pathways [20]. Initial reports suggest that the cancer cells consume a significant amount of glutamine *in vivo* as well as *in vitro* during their growth [21]. Not only the cell growth or proliferation but other aspects of cancer progression are also regulated by glutamine itself. During tumor growth, cancer cells frequently face glutamine deprivation [22]. Glutamine starvation also imparts signaling changes to the cancer cells [23], which then impacts the overall cancer progression. Previous reports suggest that glutamine is important for the cell proliferation of highly migrating cancer cells. Therefore, glutamine starvation or targeted inhibition of glutamine metabolism is a potential new strategy for combating cancer [9, 10, 24]. However, the mechanism by which glutamine or its metabolites regulate cancer growth and metastasis is not well established. Cacace *et al.* have revealed that the presence of glutamine in the medium can regulate cellular signaling and cell proliferation, even when the glutamine metabolism is inhibited [23]. But what is the impact of glutamine on the EMT or how glutamine regulates the metastatic potential of cancer cells is not well understood. In the present study, the essential role of glutamine in invasiveness and migration have been elucidated in ovarian cancer (OVCA) cells PA1 and SKOV3. The interesting fact is that glutamine starvation downregulates the invasive and migratory potential of different OVCA cells following different signaling pathways.

ETS1 has been previously described as a potential marker for ovarian cancer. It is a key regulatory factor of EMT and metastasis in ovarian cancer cells [13]. Its gain of function is related to the poor prognosis of ovarian cancer (Figure 2C). In PA1 cells, glutamine deprivation reduced the expression of ETS1 in an HIF1α-dependent manner. On contrary, in SKOV3 cells, glutamine deprivation did not reduce the expression of ETS1 but it is translocated from the nucleus to the cytoplasm in an HIF1α-independent manner. It seems that these differential ETS1 regulation strategies are due to the activation of separate pathways in these cell lines, which maintain their cancer phenotype and invasive potential. It is also possible that glutamine regulates different signaling pathways in these two cell lines. The differences in the regulation of ETS1 in different cell lines of the same cancer phenotype shows the broader aspect of tumor heterogeneity, which occurs in tumors of different origin and mutation. The final effect of glutamine starvation is similar for different cancer cells but the pathway is different. To exemplify, Jo *et al.* also (2012) reported that Bee venom (1–5 μg/ml) and melittin treatment inhibited cancer cell growth, inducing apoptosis in both PA1 and SKOV3 cells upon activating caspase-8 and caspase-3 respectively. Thus the same fate is observed following these drug treatment through the activation of different signaling pathways [25]. After treatment, apoptosis in PA1 cells is triggered by active caspase-8, while in SKOV3 cells caspase-3 activation occurs.

There could be other possible reasons too which can explain the differential ETS1 regulation in these two cell lines. It has been reported that glutamine starvation is able to upregulate the p53 protein [26] and the wild type p53 can downregulate ETS1 mRNA expression, while its mutant form cannot [27]. SKOV3 cells are p53 deficient, whereas the PA1 contains its wild type form. Thus, it is possible that upon glutamine starvation, in both the cells, the ETS1 is transported out from the nucleus, and particularly in PA1 cells, the increased p53 is downregulating the ETS1 mRNA expression. Another report depicts that the synthesis of a different ETS1 isoform (ETS1-p27), due to differential splicing, causes a reduction in DNA binding and transcriptional activity of full-length normal variant of ETS1 (ETS1-p51). This reduced DNA binding is followed by the de-localization of ETS1-p51 from the nucleus to the cytoplasm [28]. Therefore, it is even possible that in SKOV3 cells, increased ETS1-p21 isoform synthesis could result in ETS1-p51 delocalization from the nucleus to the cytoplasm.

Altogether, these data suggest that targeting glutamine transport and metabolism might give a therapeutic approach against cancers of different origin or mutation. Simultaneously studying the signaling mechanisms regulated by glutamine is very important. Because, inhibiting the metabolic function of glutamine does not reduce its concentration in blood, and hence it can also exert some metabolism-independent signaling cascades. Therefore, further research is needed to decipher the signaling mechanisms of glutamine in regulating cancer progression.

## Declarations

### Author contribution statement

Sib Sankar Roy: Conceived and designed the experiments; Analyzed and interpreted the data; Contributed reagents, materials, analysis tools or data; Wrote the paper.

Parash Prasad: Conceived and designed the experiments; Performed the experiments; Analyzed and interpreted the data; Wrote the paper.

### Funding statement

This work was supported by SERB project GAP-360 (EMR/2016/002578) and CSIR in-house projects, and Parash Prasad was supported by CSIR research fellowship (31/002[1029]/2015- EMR- I).

### Data availability statement

Data will be made available on request.

### Declaration of interests statement

The authors declare no conflict of interest.

### Additional information

No additional information is available for this paper.

## Appendix A Supplementary data

ETS1 Supplementary 26April-2021.pptx ETS1 Supplementary 26April-2021.pptx

## References

[1] Mendez M.G., Kojima S., Goldman R.D. (2010 Jun). Vimentin induces changes in cell shape , motility , and adhesion during the epithelial to mesenchymal transition. FASEB J..

[2] Lee J.J., van de Ven R.A.H., Zaganjor E., Ng M.R., Barakat A., Demmers J.J.P.G., Finley L.W.S., Herrera K.N.G., Hung Y.P., Harris I.S., Jeong S.M., Danuser G., McAllister S.S., Haigis M.C. (2018). Inhibition of epithelial cell migration and Src/FAK signaling by SIRT3. Proc. Natl. Acad. Sci. Unit. States Am..

[3] Vallenius T. (2013). Actin stress fibre subtypes in mesenchymal-migrating cells. Open Biol.

[4] Webb A.H., Gao B.T., Goldsmith Z.K., Irvine A.S., Saleh N., Lee R.P., Lendermon J.B., Bheemreddy R., Zhang Q., Brennan R.C., Johnson D., Steinle J.J., Wilson M.W., Morales-Tirado V.M. (2017). Inhibition of MMP-2 and MMP-9 decreases cellular migration, and angiogenesis in in vitro models of retinoblastoma. BMC Canc..

[5] Takai N., Miyazaki T., Nishida M., Nasu K., Miyakawa I. (2002). c-Ets1 is a promising marker in epithelial ovarian cancer. Int. J. Mol. Med..

[6] Mendez M.G., Kojima S., Goldman R.D. (2010 Jun). Vimentin induces changes in cell shape , motility , and adhesion during the epithelial to mesenchymal transition. FASEB. J..

[7] Liu C., Lin H., Tang M., Wang Y. (2015). Vimentin contributes to epithelial-mesenchymal transition cancer cell mechanics by mediating cytoskeletal organization and focal adhesion maturation. Oncotarget.

[8] Gonzalez-Moreno O., Lecanda J., Green J.E., Segura V., Catena R., Serrano D., Calvo A. (2010). VEGF elicits epithelial-mesenchymal transition (EMT) in prostate intraepithelial neoplasia (PIN)-like cells via an autocrine loop. Exp. Cell Res..

[9] Chen L., Cui H. (2015). Targeting glutamine induces apoptosis: a cancer therapy approach. Int. J. Mol. Sci..

[10] Prasad P., Ghosh S., Roy S.S. (2021). Glutamine deficiency promotes stemness and chemoresistance in tumor cells through DRP1-induced mitochondrial fragmentation. Cell. Mol. Life Sci..

[11] Yang L., Moss T., Mangala L.S., Marini J., Zhao H., Wahlig S., Armaiz-Pena G., Jiang D., Achreja A., Win J., Roopaimoole R., Rodriguez-Aguayo C., Mercado-Uribe I., Lopez-Berestein G., Liu J., Tsukamoto T., Sood A.K., Ram P.T., Nagrath D. (2014). Metabolic shifts toward glutamine regulate tumor growth, invasion and bioenergetics in ovarian cancer. Mol. Syst. Biol..

[12] Meng D., Yang Q., Wang H., Melick C.H., Navlani R., Frank A.R., Jewell J.L. (2020). Glutamine and asparagine activate mTORC1 independently of Rag GTPases. J. Biol. Chem..

[13] Ray U., Roy Chowdhury S., Vasudevan M., Bankar K., Roychoudhury S., Roy S.S. (2017). Gene regulatory networking reveals the molecular cue to lysophosphatidic acid-induced metabolic adaptations in ovarian cancer cells. Mol. Oncol..

[14] Bhattacharya R., Mitra T., Ray Chaudhuri S., Roy S.S. (2018). Mesenchymal splice isoform of CD44 (CD44s) promotes EMT/invasion and imparts stem-like properties to ovarian cancer cells. J. Cell. Biochem..

[15] Ay T., Anthony H.T., Konje J.C. (2020). Selection of endogenous control reference genes for studies on type 1 or type 2 endometrial cancer. Sci. Rep..

[16] Kato Y., Ozawa S., Tsukuda M., Kubota E., Miyazaki K., St-Pierre Y., Hata R.I. (2007). Acidic extracellular pH increases calcium influx-triggered phospholipase D activity along with acidic sphingomyelinase activation to induce matrix metalloproteinase-9 expression in mouse metastatic melanoma. FEBS J..

[17] Szklarczyk D., Gable A.L., Lyon D., Junge A., Wyder S., Huerta-Cepas J., Simonovic M., Doncheva N.T., Morris J.H., Bork P., Jensen L.J., Von Mering C. (2019). STRING v11: protein-protein association networks with increased coverage, supporting functional discovery in genome-wide experimental datasets. Nucleic Acids Res..

[18] Anaya J. (2016). OncoLnc: linking TCGA survival data to mRNAs, miRNAs, and lncRNAs. PeerJ Comput. Sci..

[19] Tam S.Y., Wu V.W.C., Law H.K.W. (2020). Hypoxia-induced epithelial-mesenchymal transition in Cancers : HIF-1 α and beyond. Front. Oncol..

[20] Coller H.A. (2014). Is cancer a metabolic disease? 9582978472. Am. J. Pathol..

[21] Yang L., Venneti S., Nagrath D. (2017). Glutaminolysis: a hallmark of cancer metabolism. Annu. Rev. Biomed. Eng..

[22] Pan M., Reid M.A., Lowman X.H., Kulkarni R.P., Tran T.Q., Liu X., Yang Y., Hernandez-Davies J.E., Rosales K.K., Li H., Hugo W., Song C., Xu X., Schones D.E., Ann D.K., Gradinaru V., Lo R.S., Locasale J.W., Kong M. (2016). Regional glutamine deficiency in tumours promotes dedifferentiation through inhibition of histone demethylation. Nat. Cell Biol..

[23] Cacace A., Sboarina M., Vazeille T., Sonveaux P. (2017). Glutamine activates STAT3 to control cancer cell proliferation independently of glutamine metabolism. Oncogene.

[24] Ratnikov B., Jeon Y.J., Smith J.W., Ronai Z.A. (2015). Right on TARGET: glutamine metabolism in cancer. Oncoscience.

[25] Jo M., Park M.H., Kollipara P.S., An B.J., Song H.S., Han S.B., Kim J.H., Song M.J., Hong J.T. (2012). Anti-cancer effect of bee venom toxin and melittin in ovarian cancer cells through induction of death receptors and inhibition of JAK2/STAT3 pathway. Toxicol. Appl. Pharmacol..

[26] Blagih J., Humpton T.J., Adams P.D., Vousden K.H., Hock A.K., Blagih J., Robertson N.A., Labuschagne C.F., Kruiswijk F. (2018). Article a role for p53 in the adaptation to glutamine starvation through the expression of SLC1A3 article a role for P53 in the adaptation to glutamine starvation through the expression of SLC1A3. Cell Metab.

[27] Dittmer J. (2003). The biology of the Ets1 proto-oncogene. Mol. Canc..

[28] Laitem C., Leprivier G., Begue A., Monte D., Larsimont D., Dumont P., Aumercier M. (2009). Ets-1 p27 : a novel Ets-1 isoform with dominant-negative effects on the transcriptional properties and the subcellular localization of Ets-1 p51. Oncogene.

