# Supplementary figures and images for: Glutamine regulates ovarian cancer cell migration and invasion through ETS1
Source: Heliyon. 2021 May 31;7(5):e07064. doi: 10.1016/j.heliyon.2021.e07064 (PMC8180613; doi:10.1016/j.heliyon.2021.e07064)

## Slide 1
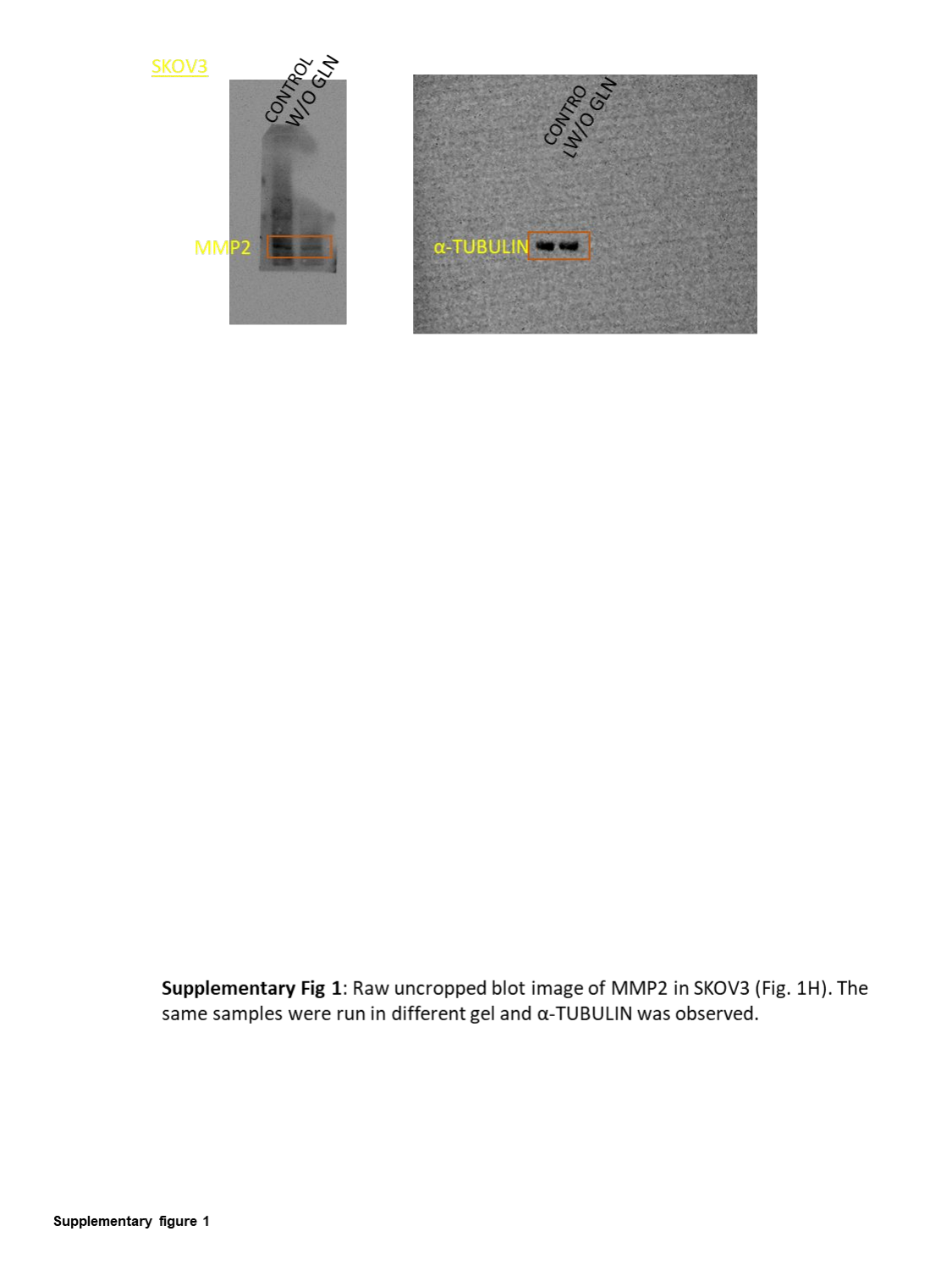

## Slide 2
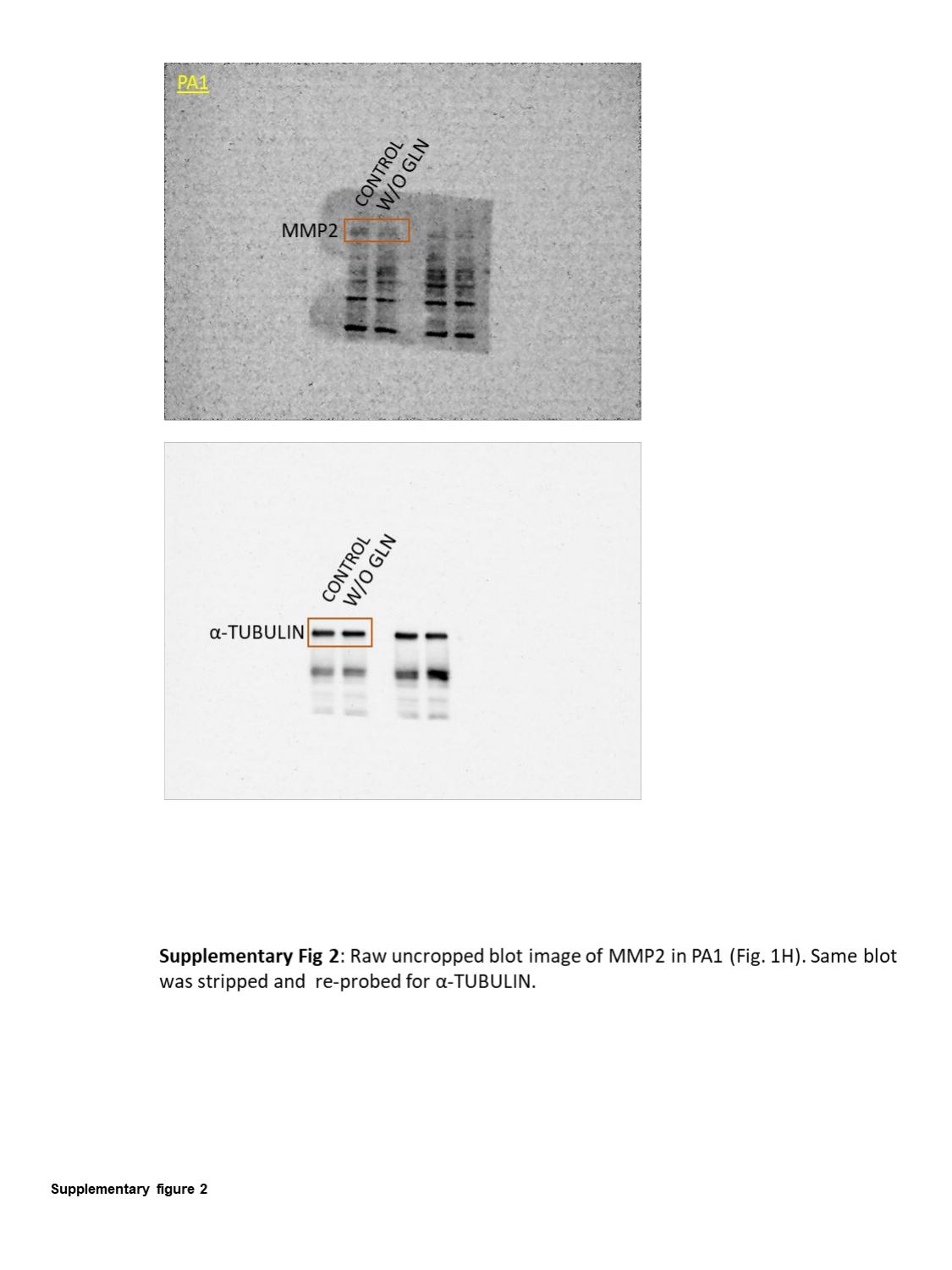

## Slide 3
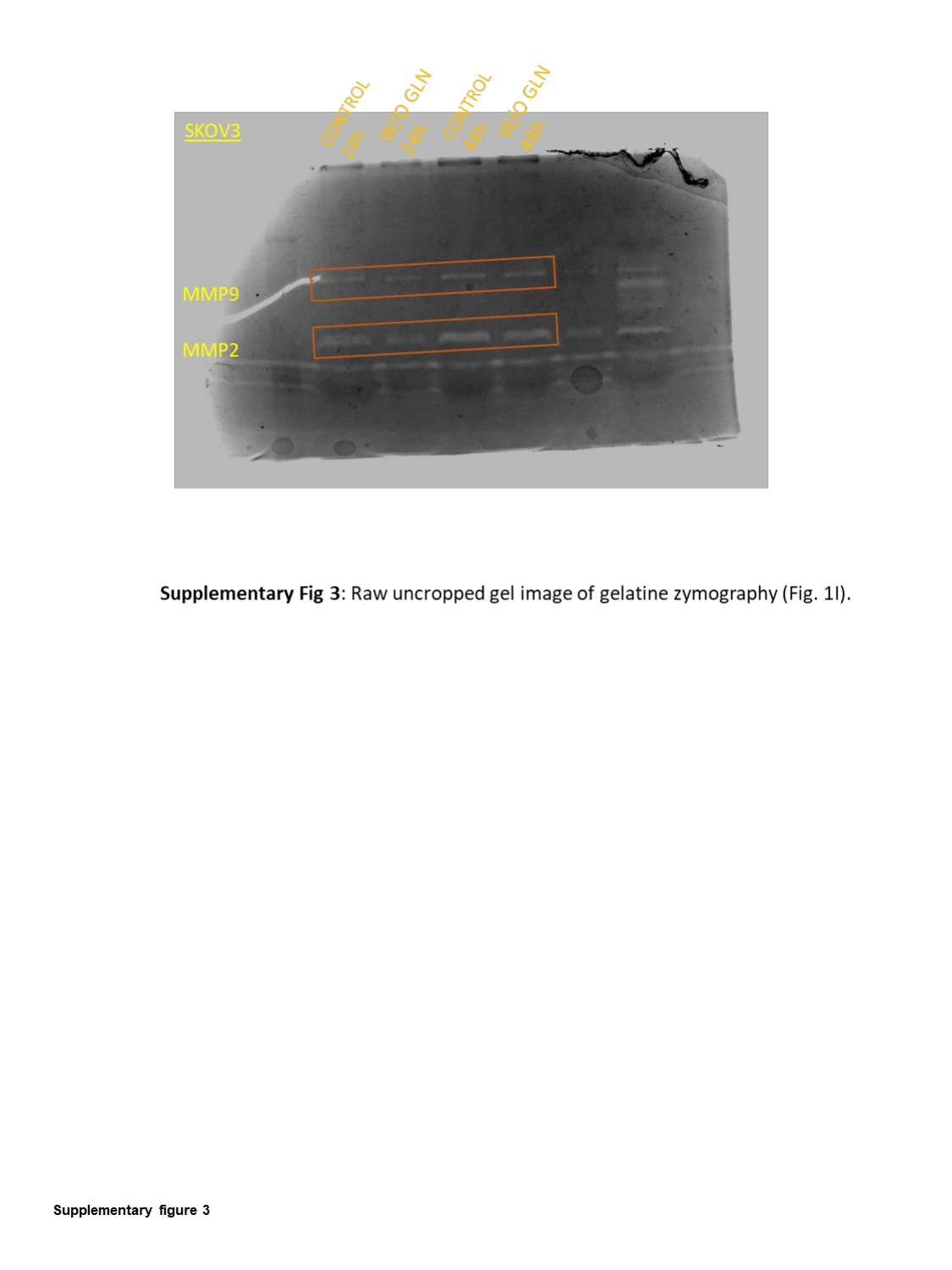

## Slide 4
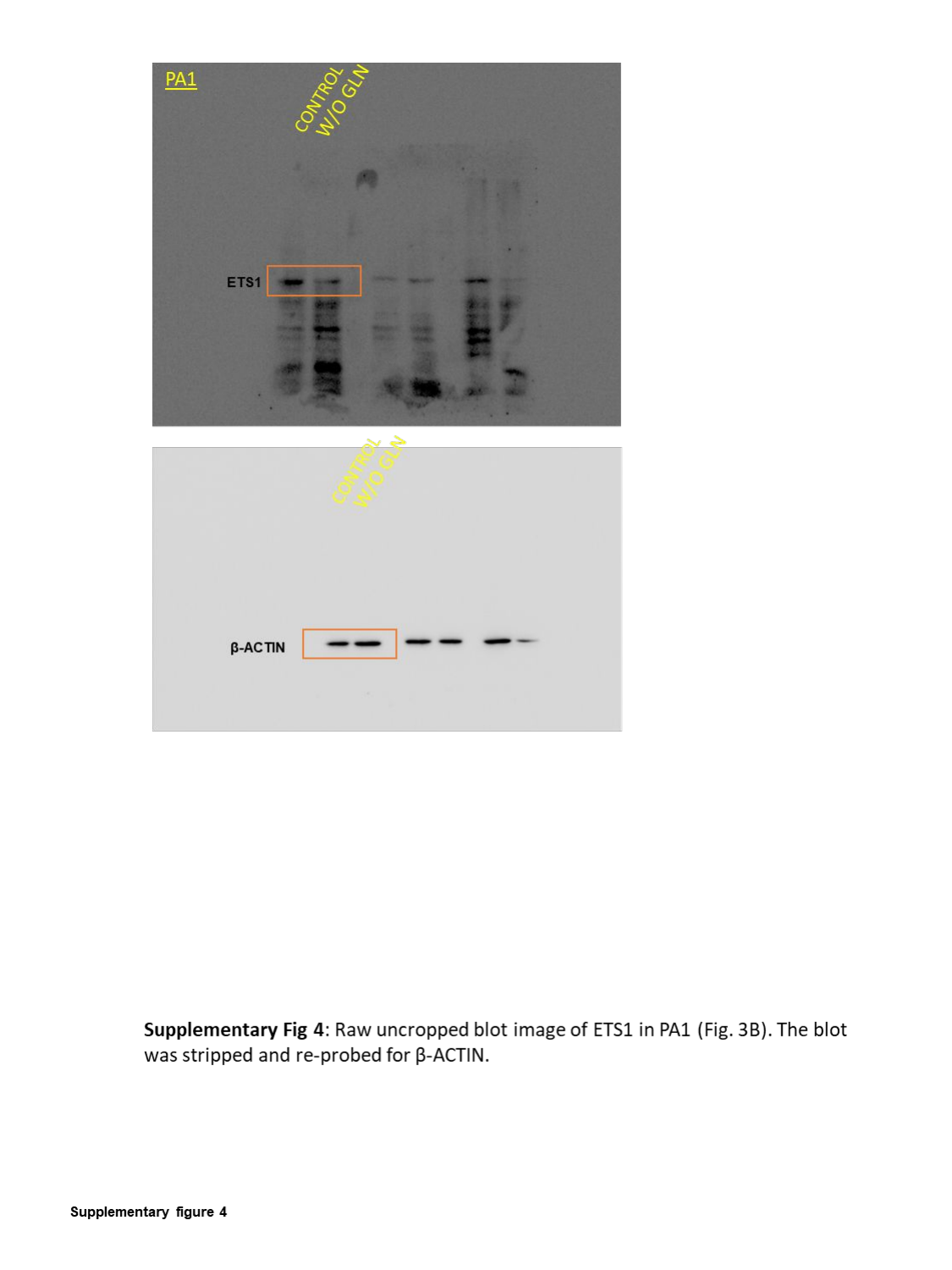

## Slide 5
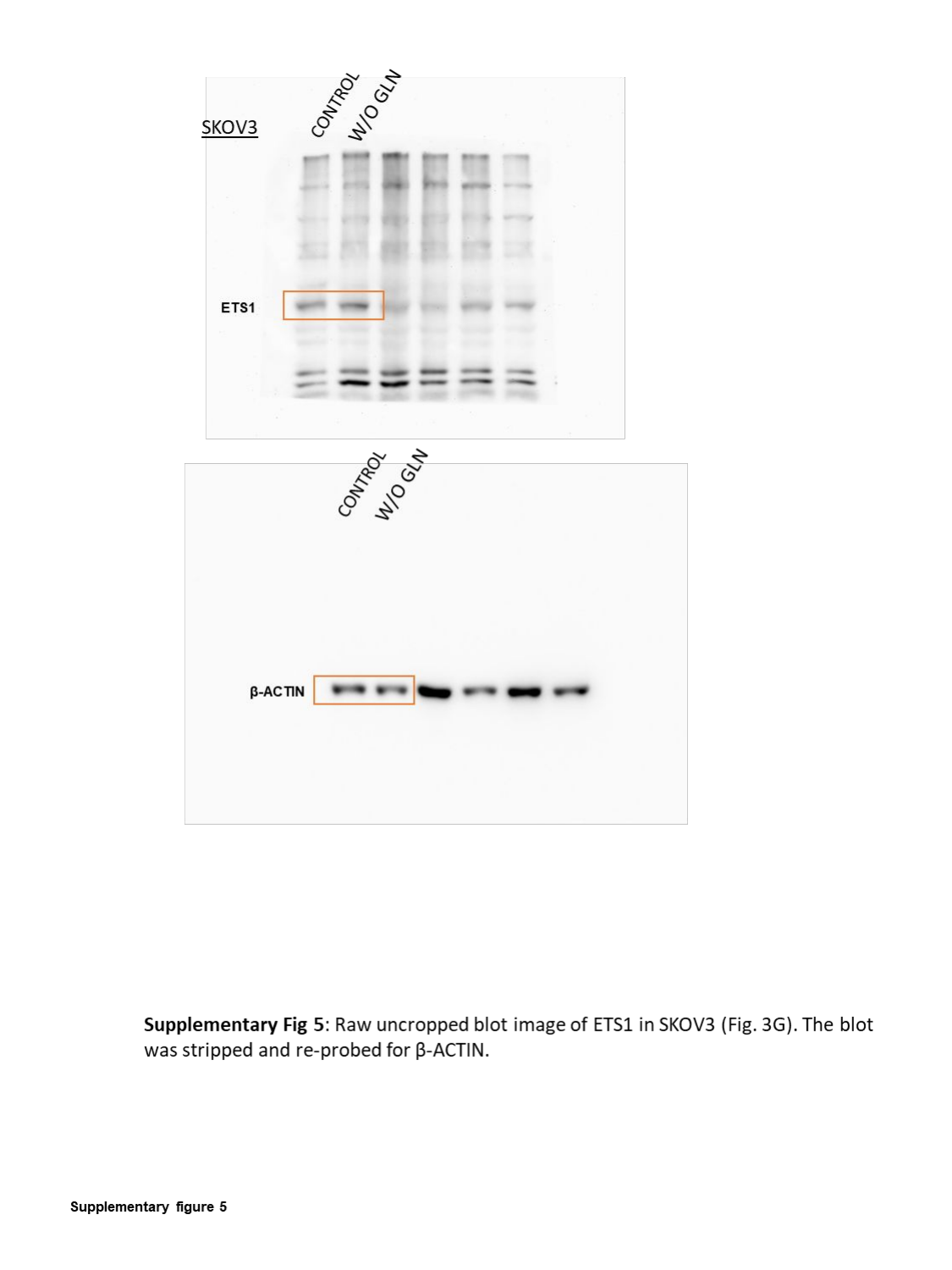

## Slide 6
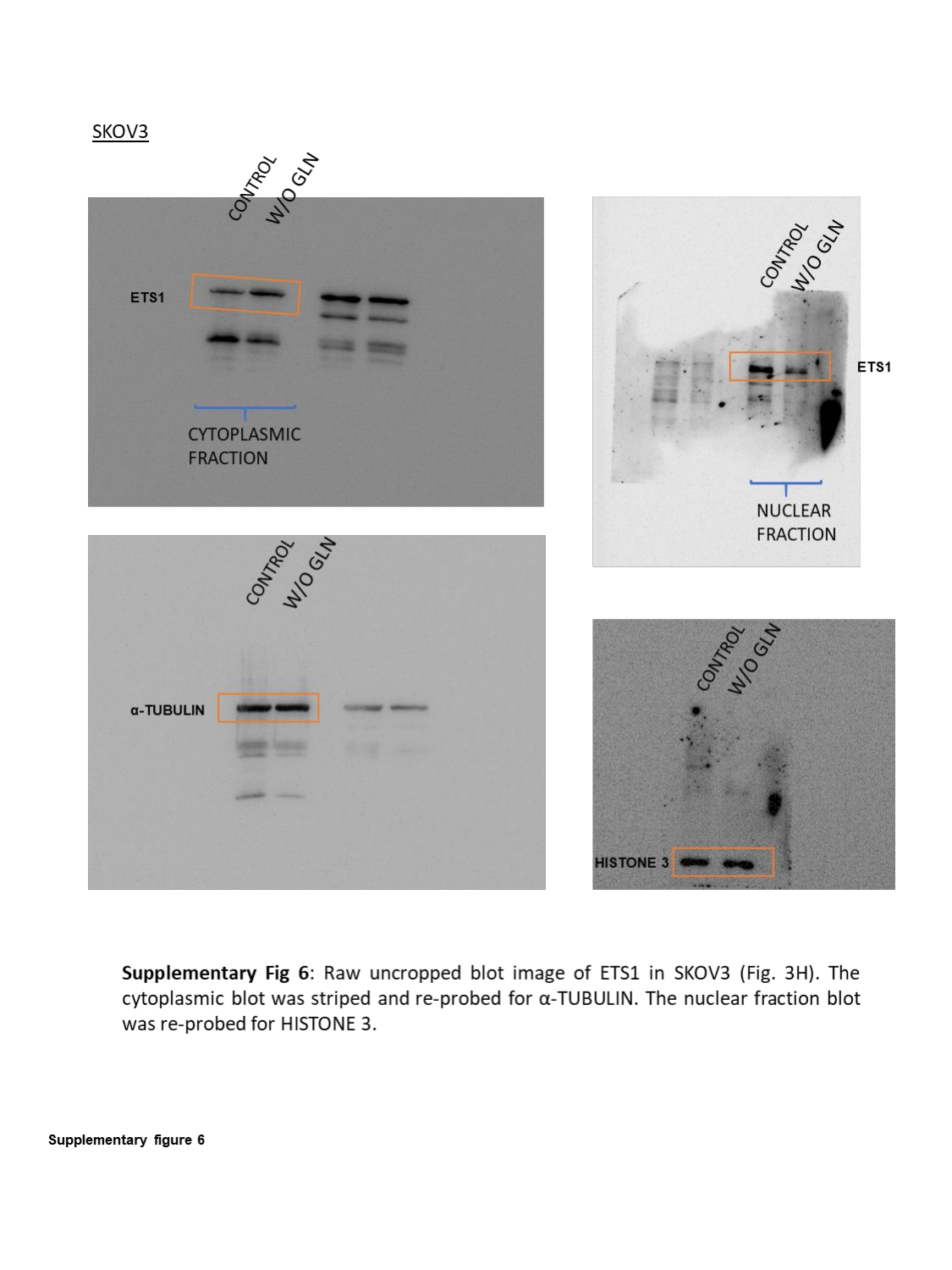

## Slide 7
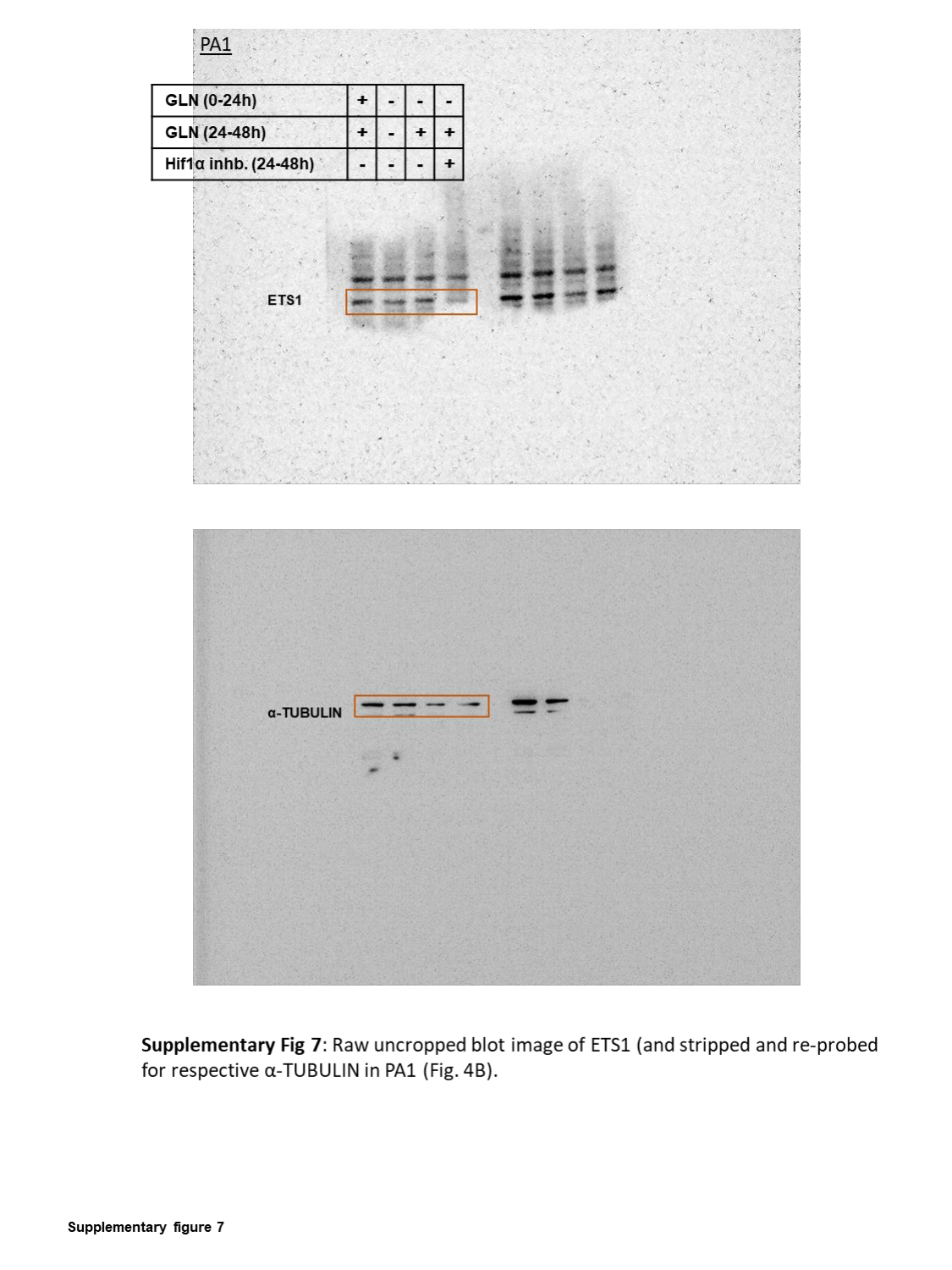

## Slide 8
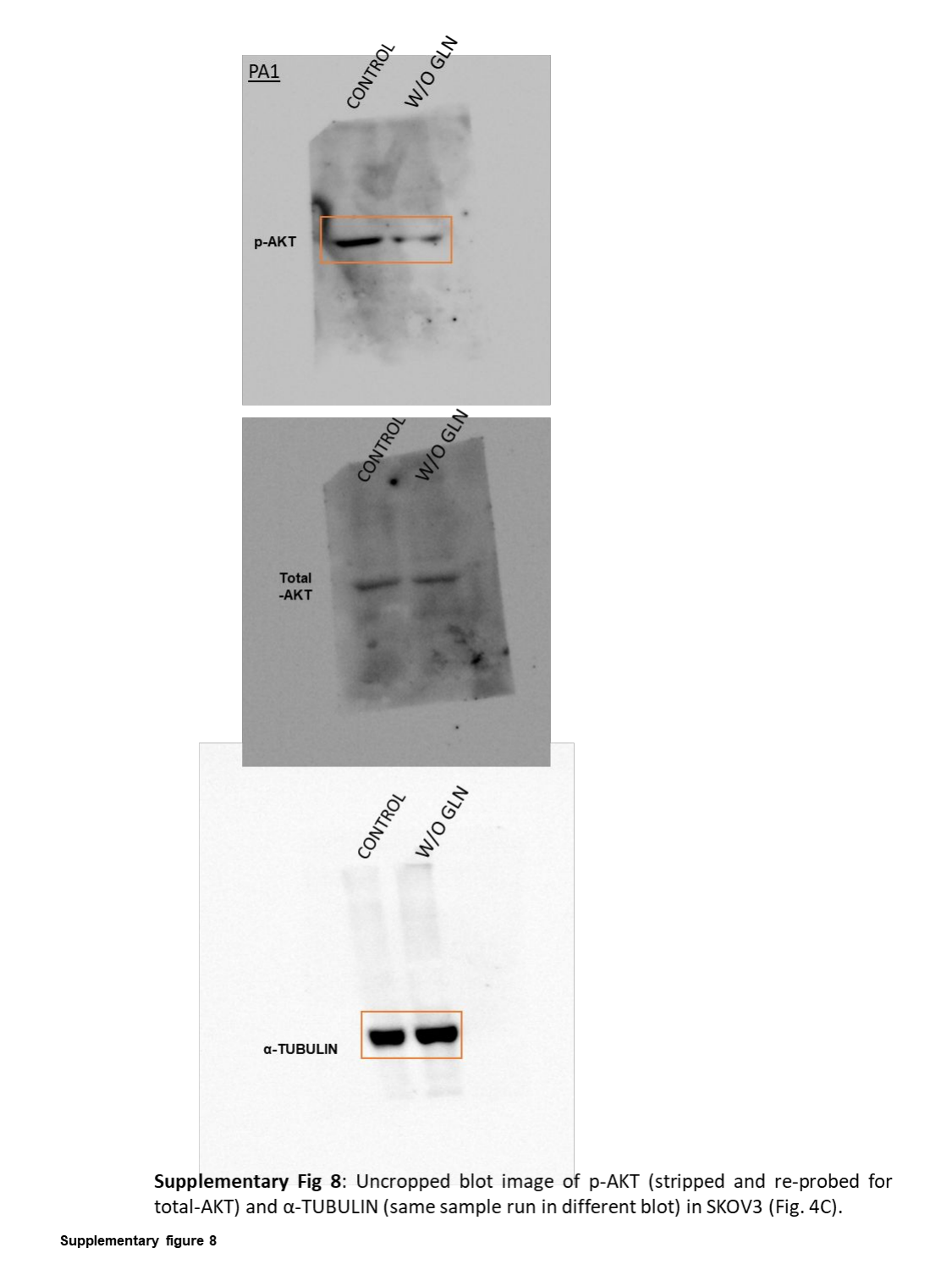

Supplement: ETS1 Supplementary 26April-2021.pptx [file mmc1.pptx]
